# New Insights into the Crystal Chemistry of FeB-Type Compounds: The Case of CeGe

**DOI:** 10.3390/ma15249089

**Published:** 2022-12-19

**Authors:** Riccardo Freccero, Emmelina Frick, Caroline Wilthorn, Julia-Maria Hübner

**Affiliations:** 1 Dipartimento di Chimica e Chimica Industriale, Università degli Studi di Genova, Via Dodecaneso 31, I-16146 Genova, Italy; 2 Centre for Analysis and Synthesis, Lund University, Naturvetarvägen 14, 223 62 Lund, Sweden

**Keywords:** intermetallic compounds, crystal chemistry, X-ray diffraction, chemical bonding

## Abstract

Several alkaline earth or rare earth binary monosilicides and -germanides possess complex bonding properties, such as polycation formation exceeding the scope of classical electron counting rules. In this study, we present characterization by powder and single-crystal diffraction and thermal analysis of CeGe, one of the few monogermanides crystallizing in the FeB-type structure. Comparative computational investigations for structure types experimentally observed for monogermanides and alternative structures with different structural motifs were performed to gain energetical insights into this family of compounds, underlining the preference for infinite germanium chains over other structural motifs. Formation enthalpy calculations and structural chemical analysis highlight the special position of FeB-type compounds among the monogermanides.

## 1. Introduction

Electron counting rules are simple but powerful schemes allowing for the explanation of electronic properties and chemical bonding or even the prediction of structural arrangements for a surprisingly large number of compounds. Whereas this approach works well for certain families among the inorganics, such as Zintl [1] or cluster compounds [2], the neighboring class of intermetallics lacks general systematics explaining their structure, properties, and bonding interrelation [3,4]. In order to disclose such relationships, combined experimental and theoretical studies, including energy and bonding calculations, have to be systematically performed.

Interestingly, such analyses have often revealed unanticipated bonding features for groups of compounds that formally follow electron counting schemes, e.g., the Zintl–Klemm concept. The latter, in its stricter interpretation, assumes the formation of homopolar two-center, two-electron bonds among p-block elements with the aim to fulfil the octet rule after a formal electron transfer from electropositive metal species. Up to now, several polyanionic fragments with intriguing structural and chemical peculiarities, often exceeding chemists’ expectations and predictions, have been reported. Among them, zig-zag chains are common for binary alkaline earth (*AE*) or rare earth metal (*RE*) silicides and germanides. Complex bonding interactions were discovered both for compounds formally fulfilling the octet rule, such as CrB-type CaSi [5], and FeB-type LuGe, wherein the formation of two-bonded germanium chains, (2*b*)Ge, formally bearing a −2 charge, leads to an ionic formulation comprising excess electrons: Lu^3+^[(2*b*)Ge^2−^] × 1 *e*^−^ [6]. 

Recently, such electrons were found to be responsible for the formation of four-atomic Lu_4_ bonds besides Lu-Ge polar interactions [6]. There are different possibilities of how these interactions can influence the resulting structure, especially the infinite germanium chains as the main structural element. Three supposable scenarios were selected and investigated using electronic structure and energetical calculations and structure chemical analysis to gain a deeper understanding of the origin of bonding interactions and their influence on structural features in FeB-type monogermanides. The chains can show (1) a Peierls distortion resulting in a superstructure formation comprising dumbbells instead of chains at low temperatures [7], (2) a deformation by changing the chain distances *d*_Ge-Ge_, or (3) a deformation combined with an inclination perpendicular to the running direction of the chains. However, only four rare-earth monogermanides with the same structure type were reported (Table 1). The compound CeGe has been known since the work of Parthé [8], but the structure of the compound (FeB-type) has only been inferred from the indexing of the powder X-ray diffraction pattern. This motivated a reinvestigation of CeGe based on single-crystal diffraction data.

## 2. Materials and Methods

Synthesis. Sample handling was performed in an argon-filled glovebox. The synthesis was conducted in an arc furnace under argon atmosphere. To ensure homogenization, each sample was melted two times. Cerium was added in excess (1.8%) to compensate for evaporation loss.

X-ray diffraction. For the analysis, the samples were prepared for powder X-ray diffraction (Stoe Stadi MP (STOE & Cie GmbH, Darmstadt, Germany), Mythen 1k detector, Cu-*K*_α_ radiation, *λ* = 1.54178 Å) in transmission mode. Single crystals were chosen and analyzed with single-crystal X-ray diffraction (Xcalibur (Rigaku Oxford Diffraction, Sevenoaks, Kent, UK), EosS2, CCD plate detector, graphite monochromator, Mo-*K*_α_ radiation, *λ* = 0.71073 Å) at room temperature and at −173 °C. Data integration, structure solution, and refinements were conducted with the programs CrysAlisPro [31], Superflip [32], and JANA2006 [33].

Thermal analysis. Differential scanning calorimetry (DSC) was performed between −90 and 100 °C in a DSC Q2000 device (T.A. Instruments, New Castle, DE, USA) with heating and cooling rates of 10 K min^−1^ (sample mass 25.568 mg) in an Al pan and lid.

Computational Details. Total energy calculations were performed at a DFT/PBE level of theory using the Quantum Espresso software package (v5.4) [34] for the following compounds: CeGe simulated with the *oP*8–FeB, *oS*8–CrB, *oS*16–LaSi, *hP*12–Na_2_O_2_, and *hP*8–Li_2_O_2_-*β* structures, YGe simulated as *oS*8–CrB and *hP*12–Na_2_O_2_, LaGe as *oS*16–LaSi and *hP*12–Na_2_O_2_, CaAs and KSe as *oP*8–FeB, *oS*8–CrB, *oS*16–LaSi, and *hP*12–Na_2_O_2_. For the sake of conciseness, such structures will just be indicated by their prototypes in the following ways. The recommended projector-augmented wave (PAW) [35] sets, available at the PSlibrary [36], were employed. The semicore states 5*s*, 5*p*, and 5*d* for Ce and La; 4*s* and 4*p* for Y; 3*s* and 3*p* for K and Ca; and 3*d* for Ge and Se were included in the valence configuration. Thus, the 4*f* states of Ce were not included in such configuration, and all calculations were non-spin polarized. The orbital occupancies at the Fermi level were treated with Gaussian smearing of 0.01 Ry. The plane-wave and density cut-off were set to 45 Ry and 450 Ry, respectively. The Brillouin zone was sampled within uniform grids (Monkhorst–Pack [37]) generated with the *k*-point meshes listed in Table 2.

The convergence threshold for self-consistency was set to 1.0∙10^−9^ Ry. The electronic density of states (DOS) for CeGe with the FeB, Na_2_O_2_, and Li_2_O_2_ type of structures were computed with the aid of the LOBSTER software (v4.1.0) [38,39,40,41], which reconstructs electronic structures through the projection of PAW-based wave functions onto atomic-like basis sets.

For this purpose, the PAW wave functions were generated by running an additional single-point calculation slightly reducing the *k*-mesh: (4, 8, 6) for FeB, (6, 6, 8) for Na_2_O_2_, and (8, 8, 4) for Li_2_O_2_. The projection was performed with the local pbeVaspFit2015 [39] basis set; Ce 5*s*, 5*p*, 5*d*, 6*s*, and Ge 3*d*, 4*s*, 4*p* orbitals were selected, leading to excellent absolute charge spilling (<0.9%). DOS curves were plotted utilizing the wxDragon software (v2.1.8) [42].

## 3. Results and Discussion

### 3.1. Crystal Structure and Thermal Analysis

CeGe, obtained by the arc-melting of the elements with a 1.8% excess of Ce, is crystallizing in space group *Pnma* with *a* = 8.3524(4) Å, *b* = 4.0852(2) Å, and *c* = 6.0322(3) Å (Table 3) in accordance with previous powder diffraction studies (Table S1, Figures S1 and S2) [8,43,44]. The structure refinement was performed on single-crystal X-ray diffraction data (Figure S3, acquired at room temperature), resulting in residuals *R* = 0.0352, *wR* = 0.0321 (Table 3, Table 4 and Table 5). 

The compound is isotypic to FeB [45]. In CeGe, each cerium atom possesses seven germanium and ten cerium neighbors (Figure S2). Germanium is coordinated by two germanium and seven cerium atoms. The crystal structure of CeGe constitutes columns of triangular prisms along [010], connected by two rectangular faces. Each prism has one shared basal edge and one common atom at the apexes opposite this basal edge with four other prisms. Within these prisms, chains of interconnected germanium atoms run along the [010] direction. The Ge-Ge chains are arranged in sheets along the (011) plane. These sheets are alternately stacked parallel to the [100] direction, in which solely Ce or Ge atoms can be observed. 

**Figure 1:**
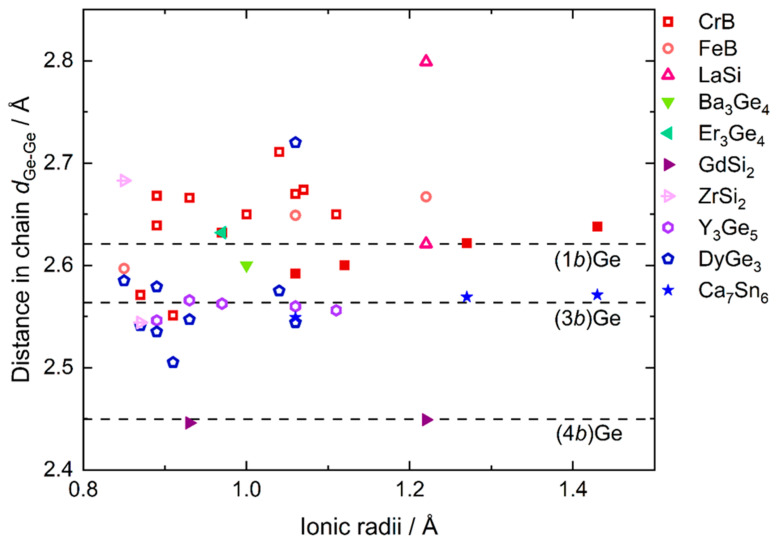
Distances *d*_Ge-Ge_ of selected compounds comprising Ge-chains [6,8,9,10,11,12,13,14,15,16,17,18,19,20,21,22,23,24,25,26,27,28,29,30,48,49,50,51,52,53,54,55,56,57,58,59,60,61,62,63,64,65,66,67] versus ionic radii (for trivalent rare earth and divalent alkaline earth metals, as well as Eu [68]), ordered by their structure types. Filled symbols denote electron-precise compounds, empty symbols compounds with non-electron precise electron balances. Distances for (4*b*)Ge (*cF*8-Ge [46]), (3*b*)Ge (NaGe [69]), and (1*b*)Ge (Li_7_Ge_2_ [70]) serve as references.

The distances *d*_Ce-Ge_ range between 3.1186(4) and 3.3403(4) Å (Table 6) and *d*_Ge-Ge_ amount to 2.6739(4) Å. In comparison to *d*_Ge-Ge_ in the element (2.450 Å [46]), the distances *d*_Ge-Ge_ are noticeably long (Figure 1), corresponding to a significantly reduced bond order of *s*_ij_ = exp($\frac{d_{1}-d_{ij}}{c}$) = 0.68 (*d*_1_: *d*_Ge-Ge_ in *cF*8 Ge [46]; *d*_ij_: *d*_Ge-Ge_ in CeGe; *c* = 0.6) [47], calculated as a first estimate using the Pauling formula which approximates bond orders varying exponentially with bond distances. 

Similar one-dimensional chains of germanium occur in compounds crystallizing with different structure types [6,8,9,10,11,12,13,14,15,16,17,18,19,20,21,22,23,24,25,26,27,28,29,30,48,49,50,51,52,53,54,55,56,57,58,59,60,61,62,63,64,65,66,67] (see Table S2), whereas chains in connection with other structural motifs are found both for electron-precise and non-electron-precise phases. When appearing in combination with other structural elements, the distances in Ge–Ge chains are mostly longer than in elemental Ge but relatively similar to distances among (3*b*)Ge within the ${Ge}_{4}^{4-}$ electron-precise tetrahedral clusters in NaGe [69] (Figure 1). Compounds with infinite chains as the only structural element are electron-precise for divalent elements such as *AE* metals and possess, in that case, interatomic distances similar to (1*b*)Ge dumbbells in Li_7_Ge_2_ [70].

Although an electron-precise electron balance could be realized for rare earth monosilicides and -germanides with structures comprising solely (1*b*)Ge dumbbells in the anionic partial structure, this connectivity scenario is only found for alkaline, *AE*, and *RE* metal silicides or germanides for non-electron-precise compositions. In the first possible scenario, a Peierls distortion of the Ge-chains resulting in the formation of a superstructure at low temperatures and, therefore, dimerization in line with an electron-precise electron balance did not occur, as evidenced by the absence of features pointing towards a phase transition during DSC experiments in the range of −90 to 100 °C (Figure S4). Additional single-crystal diffraction experiments at −173 °C confirm a structure comprising Ge-chains with equal Ge–Ge distances. 

### 3.2. Stability and Electronic Structure

Theoretical calculations were performed to explain why monogermanides are an exception among *A*- (alkali metal), *AE*-, and *RE*-tetrel (group 14 elements) compounds [71,72] realizing a structural arrangement in line with the Zintl–Klemm concept and the 8 − *N* rule. Total energy calculations, including the simulation of CeGe with the structure types FeB, CrB, and LaSi reported for the *RE*Ge compounds (for optimized structural data, see Table 7) indicate that there are no relevant energy differences (see Table 7) among them. In contrast, the simulation of CeGe in the structure types Na_2_O_2_ and *β*-Li_2_O_2_ experimentally observed, e.g., for CaAs and KSe comprising (1*b*)As^2−^ and (1*b*)Se^−^ dumbbells, revealed lower stability compared to the structure types comprising chains (Table 7). These results confirm that the formation of (1*b*)Ge^3−^ dumbbells is energetically not favored. 

The comparison of the interatomic distances of the optimized CeGe with FeB and Li_2_O_2_ structures shows that the distances in the dumbbells (*d*_Ge-Ge_ = 2.79 Å) are larger than those within the zig-zag chains (*d*_Ge-Ge_ = 2.70 Å), suggesting a higher electrostatic repulsion among the (1*b*)Ge^3−^ species. For CeGe simulated in the Na_2_O_2_ structure type, two kinds of Ge dumbbells with *d*_Ge1–Ge1_ = 2.68 Å and *d*_Ge2–Ge2_ = 2.53 Å were observed. Such a difference was reported neither for CaAs [73] nor for the Na_2_O_2_ [74] prototype. Hence, although the hexagonal symmetry is retained, this observation for CeGe (Na_2_O_2_) is probably caused by more complex factors.

**Figure 2:**
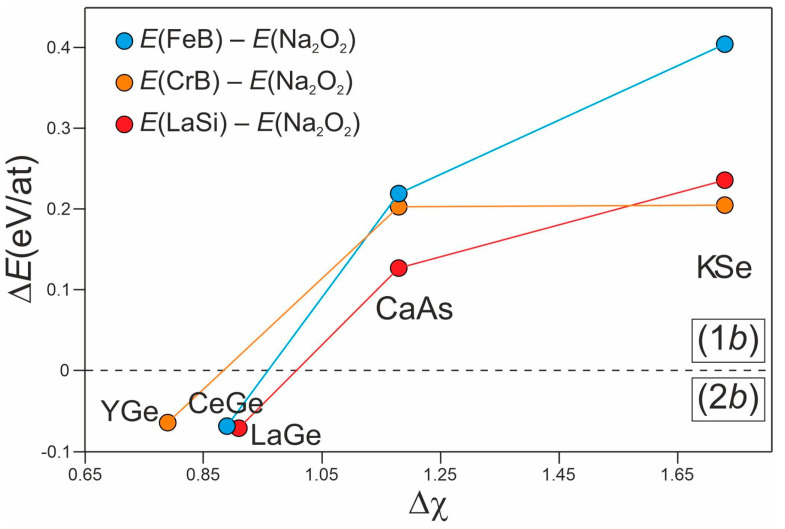
Energy difference (Δ*E*) as a function of the Pauling electronegativity difference (Δ*χ*) among the constituents of CeGe, YGe, LaGe, CaAs, and KSe. The dashed black line corresponds to Δ*E* = 0 and separates the stability region of (2*b*) polyanions, Δ*E* < 0, from that of the (1*b*) ones, Δ*E* > 0.

One reason for the preference of (2*b*)Ge chains could be found in the electronegativity difference among the constituents (Δ*χ*), which is not high enough to allow for an almost complete charge transfer of three valence electrons from Ce to Ge so that only two are formally transferred. The remaining one is probably participating in Ce–Ce interactions. Since the formation of dumbbells is disfavored along the series of the rare-earth monogermanides, YGe and LaGe have been included in this analysis as representative for the CrB and LaSi types, respectively. To prove this assumption, CaAs and KSe total energies have also been calculated in the FeB, CrB, LaSi, and Na_2_O_2_ structures and analyzed together with Δ*χ*. For CaAs and KSe, the Na_2_O_2_ modification is reported to be the most stable one. Thus, the selected *RE*Ge (*RE* = Y, La, Ce) have also been simulated with the Na_2_O_2_ structure, i.e., comprising (1*b*)Ge dumbbells. Energy differences (Δ*E*) have been calculated according to the following formulae: *E*(FeB)–*E*(Na_2_O_2_); *E*(CrB)–*E*(Na_2_O_2_); *E*(LaSi)–*E*(Na_2_O_2_). Thus, if Δ*E* < 0, the FeB/CrB/LaSi type of structure is favored. Otherwise, the Na_2_O_2_ one is expected to be most stable. The results of such an analysis are reported in Figure 2. Focusing on the blue circles in Figure 2, which indicate the energy difference between the FeB and Na_2_O_2_ structure types, one can see that, at low Δ*χ* value corresponding to the CeGe compounds, a negative Δ*E* is obtained. The aforementioned equation indicates that CeGe is most stable with the FeB structure; thus, (2*b*)Ge chains are preferred with respect to (1*b*)Ge dumbbells. Following the blue line to the right, the Δ*χ* is gradually increased until reaching first CaAs and then KSe. As clearly visible, in both cases Δ*E* > 0, indicating that these compounds are more stable when simulated in the Na_2_O_2_ type than in the FeB one, well in line with experimental results. The same trend is realized for all of the considered *RE*Ge phases (red and orange circles in Figure 2), displaying that a gradual increase of Δ*χ*, i.e., of charge transfer from metal species to the *p*-block elements, stabilizes the dumbbells-containing Na_2_O_2_ structure with respect to the other types. The only exception in this trend is represented by KSe, which displays the same *E*(CrB)–*E*(Na_2_O_2_) of CaAs (orange dots and line in Figure 2). Such anomaly is caused by the crystal structure obtained for KSe after the relaxation procedure that comprises Se dumbbells, even though the input structure was CrB. Nevertheless, the energy difference is still in favor of the Na_2_O_2_ structure.

**Figure 3:**
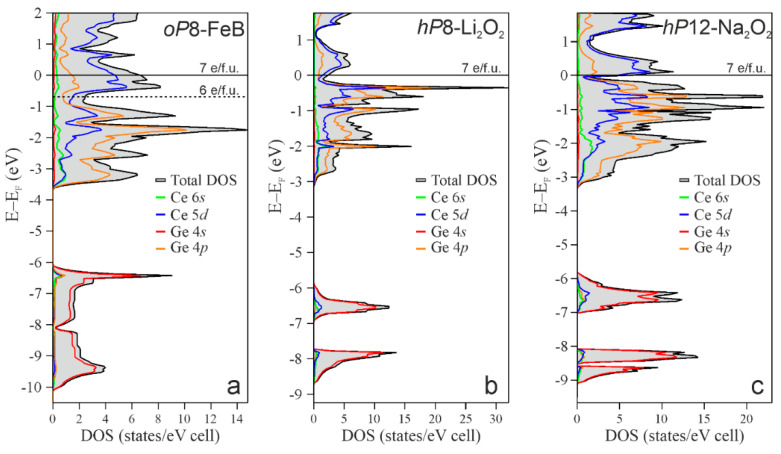
Total and orbital-projected electronic density of states for CeGe with the *oP*8-FeB structure (**a**) and simulated as *hP*8–Li_2_O_2_ (**b**) and as *hP*12–Na_2_O_2_ (**c**).

Furthermore, the electronic density of states (DOS) for CeGe in the FeB, Li_2_O_2_, and Na_2_O_2_ modifications have been calculated (see Figure 3) to give more insight into the chemical reasons behind these experimental and energetical outcomes. 

Calculated DOS curves indicate in all cases a metal-like behavior but with a noticeable difference: Li_2_O_2_ (Figure 3b) and Na_2_O_2_ (Figure 3c) show a pseudogap at the Fermi level, as typically expected for metallic germanides formally accomplishing the Zintl formalism [75,76,77]. Conversely, the pseudogap in the DOS of CeGe (FeB) occurs at approximately −0.6 eV (Figure 3a), so that the Fermi level lies in a region with a high density of states. The DOSs can be conveniently separated into two regions. The first one, located below −5 eV, is essentially dominated by the 4*s* states of Ge. The second one, in the −4 and −0.6 eV energy range for CeGe (FeB) and between −4 and 0 eV for CeGe (Li_2_O_2_ and Na_2_O_2_), is mainly contributed by the Ge 4*p* states that energetically overlap with the 5*d* of Ce. This finding indicates the incomplete ionization of Ce and the consequent formation of polar covalent interaction between Ce and Ge, analogously to other binary and ternary rare-earth germanides, such as LuGe [6], LuGe_3_ [65], *RE*_2_*M*Ge_6_ [75,78], *RE*_2_Pd_3_Ge_5_ [77]. In fact, in the ideal case of a complete ionization, Ce 5*d* states are expected to be empty, i.e., located above the *E*_F_. The CeGe (FeB) DOS has a third region, between about −0.6 eV and *E*_F_, in which the contribution of the 5*d* states of Ce exceeds that of Ge 4*p*. The integral of the DOS in this region gives 1 *e*/f.u. (electron per formula unit) (Figure 3a). This situation is very similar to the scenario observed in LuGe [6] wherein the “excess” electrons in the region between the pseudogap and *E*_F_ were responsible for the formation of Lu_4_ 4-atomic bonds. Similarly, the states in the third DOS region of CeGe could contribute to the formation of cerium–cerium bonding. This kind of interaction does not occur in the Li_2_O_2_ and Na_2_O_2_ type of structures.

### 3.3. Crystal-Chemical Insights Based on Bonding Considerations

**Figure 4:**
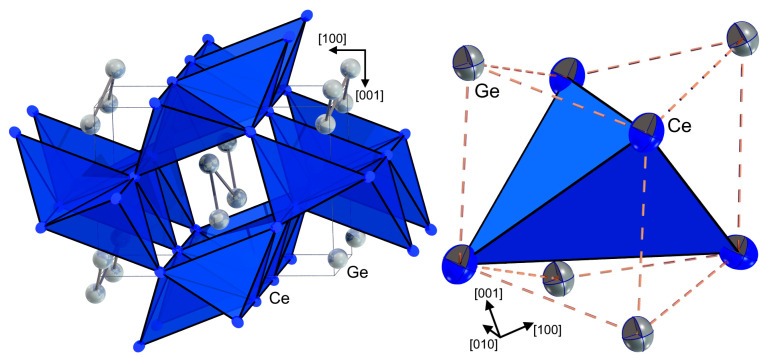
Crystal structure of CeGe depicted as (**left**) network of corner-sharing Ce_4_ tetrahedra and (**right**) stella quadrangula formed around each Ce_4_ tetrahedron.

The crystal and the electronic structure analogies observed between CeGe and LuGe allow assuming that the *RE–RE* interactions are realized within the distorted *RE*_4_ tetrahedra evidenced for the Lu-analogue [6]. Considering this chemical feature, the structure can be alternatively described as a network of corner-sharing *RE*_4_ tetrahedra with Ge chains running in the channels within the tetrahedra network (Figure 4, left). The displacement ellipsoid of the Ce atoms shows a flattening towards the channels. The displacement of Ge is slightly larger in the [001] direction, and the ellipsoid shows an elongation within the 2-D planes perpendicular to the running direction of the chains (Figure 4) that is directed towards the center of the nearest *RE*_4_ tetrahedron. Each tetrahedron is capped by four Ge atoms, resulting in a stella quadrangula (Figure 4, right). 

**Figure 5:**
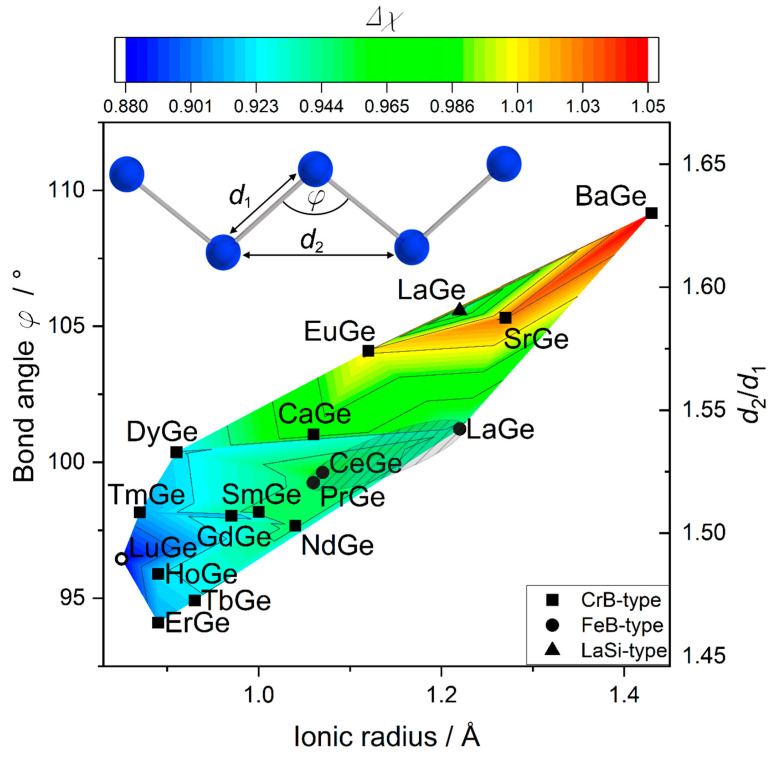
Relation between bond angle in the chains *φ*, ratio of the chain distances *d*_2_/*d*_1_ and difference in electronegativity (filled symbols—ambient pressure, open symbols—high-pressure synthesis). Striped ellipsoid marks the narrow field of ambient-pressure FeB-type rare-earth germanides.

Thus, the *RE*_4_ bonding interaction seems to influence the Ge chains significantly. The chains can react by deformation and, therefore, cause a change in the chain distances *d*_Ge-Ge_ and bond angles *φ* (Figure 5). For a more comprehensive picture of monogermanides, such analysis was extended also to germanides with the CrB type structures. Furthermore, the FeB type structure also offers the possibility of combining a deformation with an inclination perpendicular to the running direction of the chains (in [100] direction). 

The next-neighbor distances *d*_1_ with dependence on the ionic radii (for trivalent rare earth and divalent alkaline earth metals, as well as Eu [68]) remain almost constant along the series of alkaline earth and rare-earth monogermanides (Figure 1), whereas both the distance *d*_2_ after the next neighbor and bond angle increase with ionic radius. Therefore, the bond angles evolve in the same manner as the ratio *d*_2_/*d*_1_ (Figure 5), denoting that the Ge–Ge chains adjust to the size of the cation via the bond angle (and the related distance after the next *d*_2_) instead of the next-neighbor distance *d*_1_. Whereas a variety of compounds with a large range of bond angles, ionic radii, and electronegativity differences realize the CrB-type structure, FeB-type rare-earth monogermanides cover only a small range of bond angles and electronegativity differences (Figure 5, striped ellipsoid). Only the high-pressure compound LuGe does not fall into this small field. With the increasing size of the ion, the bond angle increases, underlining the high flexibility of Ge–Ge chains as a function of the cationic dimensions, probably allowing the retention of the *RE*–Ge or *AE*–Ge polar interactions that have been already described for some representatives [6,75] and also deduced from DOS calculations for CeGe. 

**Figure 6:**
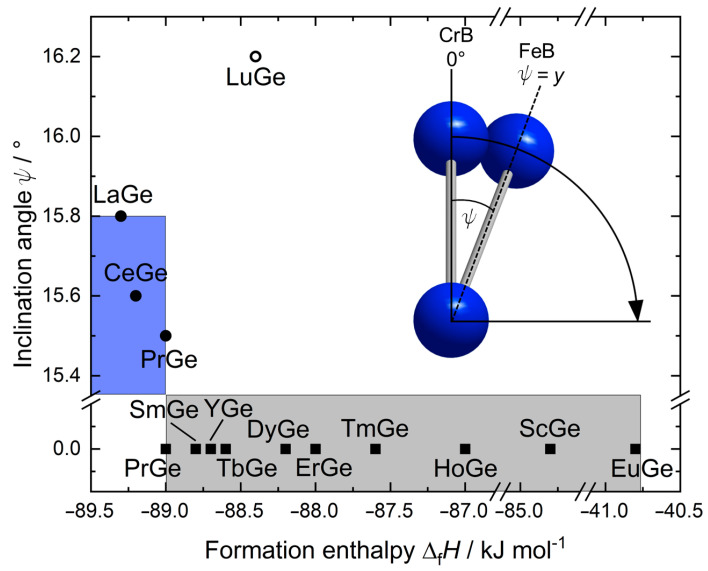
Inclination angle *ψ* of the chains vs. the enthalpy of formation Δ*_f_H*. Squares indicate CrB-type and circles FeB-type compounds. Blue and grey regions indicate fields pertaining to the FeB- and CrB-types, respectively.

To evaluate the influence of the inclination of the Ge–Ge chains as an additional degree of freedom for FeB-type compounds, the inclination angle (*ψ*) of the chains (Figure 6) was calculated from experimental data [6,13,20,21,23,24,25,26,27,28,29,30]. The inclination angle seems to mostly depend on the ionic radius (for trivalent rare earth and divalent alkaline earth metals, as well as Eu [68]), a deviation from the vertical (inclination in [100] direction, *ψ* ≠ 0°) is indeed observed only for the light rare-earth elements. The formation enthalpies Δ_f_*H,* calculated by the Miedema method [79], change according to the lanthanide contraction. The plot of the formation enthalpies Δ_f_*H* versus the inclination angle revealed that FeB- and CrB-type compounds are distributed in two fields, whereas low energy goes along with higher inclination for FeB-type compounds. For less negative energies, the CrB-type structure is realized; therefore, no inclination of the chains is observed. The dimorphic compound PrGe crystallizing in both the FeB- and the CrB-type structure is energetically located at the transition between both fields. Again, the high-pressure compound LuGe does not fall into those ranges.

## 4. Conclusions

Despite the power of simple electron counting rules, such as the Zintl–Klemm concept, interesting bonding interactions can hide even within widely “countable” compounds. Total energy and electronic structure calculations evidence the preference for complex structural interactions in contrast to what is expected from this simple scheme. Cation–cation interactions occurring in addition to classical two-center, two-electron (2*c*-2*e*) bonds in the anionic partial structure influence the overall structure. The resulting structural adaptions and the special position of FeB-type compounds among other monogermanides were traced by formation enthalpy calculations and structural chemical analysis.

Calculated DFT total energies have been related to the electronegativity difference among the constituents, used in first approximation as a measure of the effective charge transfer from the metal species to the *p*-block elements, clearly showing the key role of such a parameter in the formation of structures that fulfill, like KSe and CaAs, and do not fulfill, as *RE*Ge compounds, the Zintl rules.

For a deeper understanding, similar analyses and further chemical bonding calculations are planned for various chain compounds.

## Figures and Tables

**Table 1:** Overview of structure types of known binary monosilicides and –germanides with alkaline and rare earth elements comprising infinite chains (*C*_1_—(*oP*8)FeB, *C*_2_—(*oS*8)CrB, *C*_3_—(*oS*16)LT-LaSi) or isolated polyanions (*P*—(*oI*40)SrSi). Availability of full structure refinement data indicated by blue letters [6,8,9,10,11,12,13,14,15,16,17,18,19,20,21,22,23,24,25,26,27,28,29,30].

	Mg	Ca	Sr	Ba	Sc	Y	La	Ce	Pr	Nd	Sm	Eu	Gd	Tb	Dy	Ho	Er	Tm	Yb	Lu
**Si**		***C*_2_**	***C*_2_**	***C*_2_**	***C*_2_**	***C*_2_**	***C*_1_**	***C*_1_**	***C*_1_**	***C*_1_**	***C*_1_**	***C*_2_**	***C*_1_**	***C*_1_**	***C*_1_**	***C*_1_**	***C*_1_**	***C*_2_**	***C*_2_**	***C*_2_**
			***P***				***C*_3_**								***C*_2_**	***C*_2_**	***C*_2_**			
**Ge**		***C*_2_**	***C*_2_**	***C*_2_**	***C*_2_**	***C*_2_**	***C*_1_**	***C*_1_**	***C*_1_**	***C*_2_**	***C*_2_**	***C*_2_**	***C*_2_**	***C*_2_**	***C*_2_**	***C*_2_**	***C*_2_**	***C*_2_**		***C*_1_**
			***P***				***C*_3_**		***C*_2_**											

**Table 2:** *k*-point meshes employed to perform total energy calculations for the selected crystal structures.

Crystal Structure	*k*-Mesh
*oP*8–FeB	(6, 14, 10)
*oS*8–CrB	(12, 4, 14)
*oS*16–LaSi	(12, 4, 8)
*hP*12–Na_2_O_2_	(8, 8, 10)
*hP*8–Li_2_O_2_-*β*	(10, 10, 6)

**Table 3:** X-ray diffraction data for CeGe. Further details on the crystal structure investigations can be obtained from the FachinformationszentrumKarlsruhe, 76344 Eggenstein-Leopoldshafen, Germany (email: crysdata@fiz-karlsruhe.de, http:///www.fiz-karlsruhe.de/request_for_deposited_data.html) on quoting the depository numbers CSD-2213844.

Composition	CeGe
Space group, Pearson symbol	*Pnma* (No. 62), *oP*8
Unit cell parameters	
*a* [Å]	8.3524(4)
*b* [Å]	4.0852(2)
*c* [Å]	6.0322(3)
*V* [Å^3^]	205.86(2)
Formula units *Z*	4
Diffractometer	Rigaku Xcalibur 3, CCD detector, graphite monochromator, Mo *Kα* radiation, *λ* = 0.71073 Å
Reflections collected/independent within *F* > 4σ(*F*)	3250/303
Measurement range	−10 ≤ *h* ≤ 11, −5 ≤ *k* ≤ 5, −8 ≤ *l* ≤ 8
Fourier difference *ρ_min_*/*ρ_max_* (electrons/Å^3^)	−6.56/5.58
Residuals and GOF	*R* = 0.0352, *wR* = 0.0321, GOF = 1.37

**Table 4:** Position and displacement parameters for CeGe.

Atom	Site	*a*/x	*b*/y	*c*/z	*U*_ani_
Ce	4*c*	0.1806(1)	0.25	0.6164(2)	0.0077(3)
Ge	4*c*	0.0372(2)	0.25	0.1334(3)	0.0104(6)

**Table 5:** Components of the anisotropic displacement tensor *U* for CeGe.

Atom	*U*_11_	*U*_22_	*U*_33_	*U*_12_	*U*_13_	*U*_23_
Ce	0.0073(5)	0.0084(5)	0.0073(5)	0	0.0013(4)	0
Ge	0.010(1)	0.009(1)	0.012(1)	0	0.0003(8)	0

**Table 6:** Selected interatomic distances in CeGe.

Atom	Distance/Å	Atom	Distance/Å
Ce-2 Ge	3.1186(4)	Ge-2 Ge	2.6739(4)
Ce-2 Ge	3.1232(4)	Ge-2 Ce	3.1186(4)
Ce-1 Ge	3.1495(3)	Ge-2 Ce	3.1232(4)
Ce-1 Ge	3.3358(4)	Ge-1 Ce	3.1495(3)
Ce-1 Ge	3.3403(4)	Ge-1 Ce	3.3358(4)
Ce-4 Ce	3.8222(5)	Ge-1 Ce	3.3403(4)
Ce-2 Ce	3.9028(5)		
Ce-2 Ce	4.0852(7)		

**Table 7:** Experimental and calculated unit cell parameters and total energy differences (Δ*E*) for different CeGe polymorphs, simulated to crystallize with the FeB, CrB, LaSi, *β*-Li_2_O_2_, and Na_2_O_2_ structure types. The Δ*E* values are calculated with respect to the *oP*8–FeB structure, experimentally found to be the most stable one: *E*(FeB)–*E*(CrB); *E*(FeB)–*E*(LaSi); *E*(FeB)–*E*(*β*-Li_2_O_2_); *E*(FeB)–*E*(Na_2_O_2_).

	Experimental	Calculated				
Ge Fragments	(2*b*)Ge	(2*b*)Ge			(1*b*)Ge	
Pearson Symbol Prototype	*oP*8 FeB	*oP*8 FeB	*oS*8 CrB	*oS*16 LaSi	*hP*8 *β*-Li_2_O_2_	*hP*12 Na_2_O_2_
*a* (Å)	8.3529(5)	8.4324	4.5573	4.5402	4.7252	8.0172
*b* (Å)	4.0878(3)	4.1185	11.275	13.757	4.7252	8.0172
*c* (Å)	6.0346(3)	6.0816	4.1198	6.7556	12.2202	6.3456
Δ*E* (eV/at)	-	0.000	0.000	0.002	−0.101	−0.068

## Data Availability

CCDC 2213844 contains the supplementary crystallographic data for this paper. These data can be obtained free of charge via www.ccdc.cam.ac.uk/data_request/cif, or by emailing data_request@ccdc.cam.ac.uk or by contacting The Cambridge Crystallographic Data Centre, 12 Union Road, Cambridge CB2 1EZ, UK; fax: +44 1223 336033.

## Supplementary Materials

The following supporting information can be downloaded at: https://www.mdpi.com/article/10.3390/ma15249089/s1, Table S1: Comparison of lattice parameters of CeGe. Figure S1: Powder diffraction pattern of one obtained sample. Figure S2: Crystal structure of CeGe. (left) Unit cell with Ge-chains running along [010] direction. (right) Coordination environment of Ce and Ge, respectively. Figure S3: Diffraction pattern of CeGe with reciprocal lattice reconstructions of (a) (*hk*0), (b) (*h*0*l*), and (c) (0*kl*) layers. Figure S4: DSC, 1,3-first and second cooling, 2,4-first and second heating. Note the absence of any indication of a phase transformation in the interval measured. Table S2: Binary alkaline earth and rare earth metal germanides comprising Ge-polyanions. Average distances are given for chain motifs. References [6,8,10,12,13,20,21,23,25,26,27,28,29,30,43,44,48,49,50,51,52,53,54,55,56,57,58,59,60,61,62,63,65,66,67] are cited in the supplementary materials.
