# Supplementary material for: New Insights into the Crystal Chemistry of FeB-Type Compounds: The Case of CeGe
Source: Materials (Basel). 2022 Dec 19;15(24):9089. doi: 10.3390/ma15249089 (PMC9784137; doi:10.3390/ma15249089)
Supplement: Supplementary file 1 [file materials-15-09089-s001.zip › materials-2068093-supplementary.pdf]

Supplementary Material

New Insights into the Crystal Chemistry of FeB-Type Compounds: The Case of CeGe

Riccardo Freccero [1,*], Emmelina Frick [2], Caroline Wilthorn [2] and Julia-Maria Hübner [2,*]

1 Dipartimento di Chimica e Chimica Industriale, Università degli Studi di Genova, Via Dodecaneso 31, I-16146 Genova, Italy
2 Centre for Analysis and Synthesis, Lund University, Naturvetarvägen 14, 223 62 Lund, Sweden
* Correspondence: riccardo.freccero@unige.it (R.F.); julia-maria.hubner@chem.lu.se (J.-M.H.)

1. Crystallographic Data from Powder X-ray Diffraction Data

Table S1. Comparison of lattice parameters of CeGe.

Sample type	*a* / Å	*b* / Å	*c* / Å	Reference	Contained Phases
Powder	8.354	4.082	6.033	[1]	
Powder	8.337	4.061	6.045	[2]	
Powder	8.355	4.078	6.023	[3]	
Powder	8.3524(4)	4.0852(2)	6.0322(3)	This work, 293 K (Figure S2))	82.9% CeGe, 1.7% Ge, 3.0% CeO_2, 12.4% Ce_5Ge_4
Single crystal	8.354(4)	4.073(2)	6.029(3)	This work, 100 K	CeGe

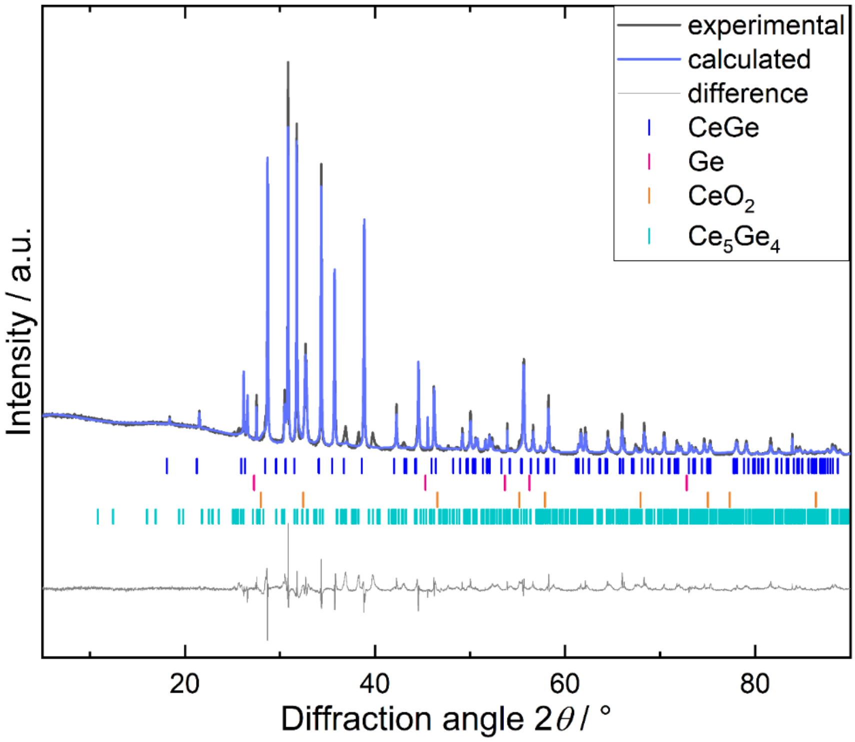

Figure S1. Powder diffraction pattern of one obtained sample.2. Crystallographic data from single crystal X-ray diffraction data.

2. Crystallographic data from single crystal X-ray diffraction data

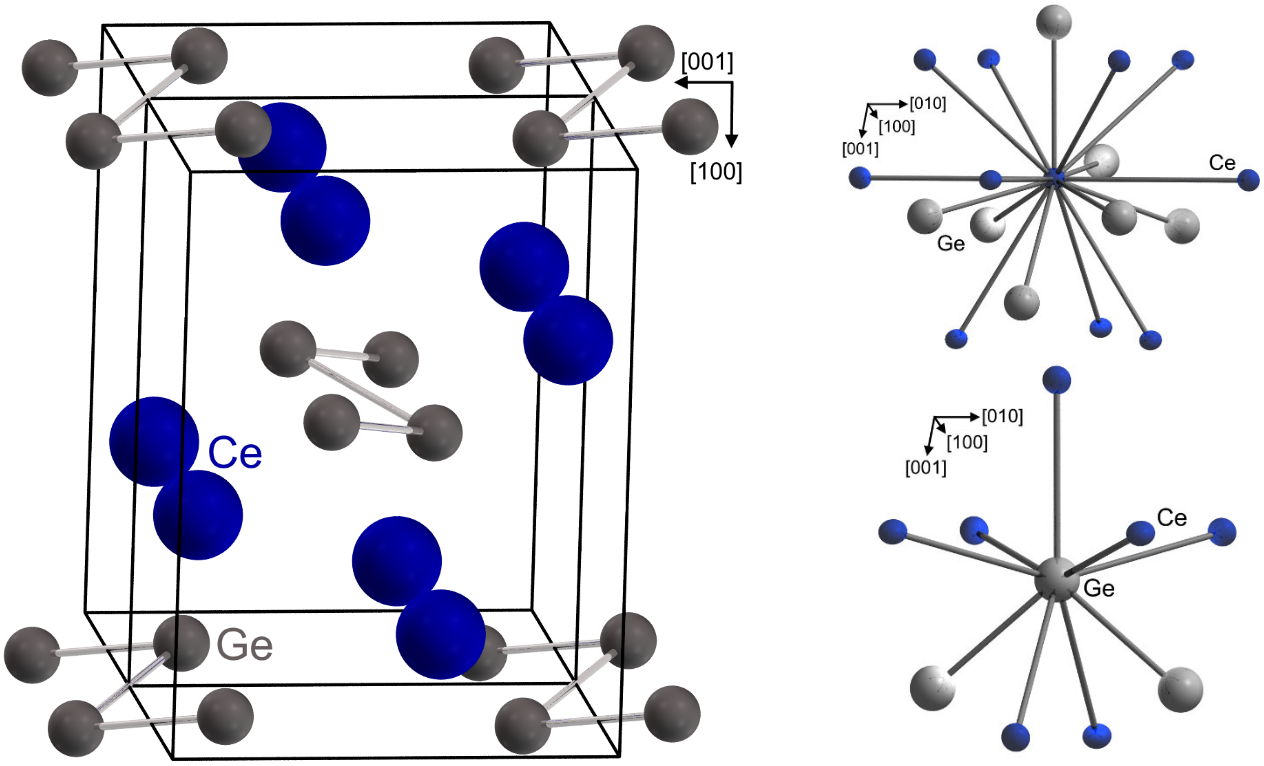

Figure S2. Crystal structure of CeGe. (left) Unit cell with Ge-chains running along [010] direction. (right) Coordination environment of Ce and Ge, respectively.

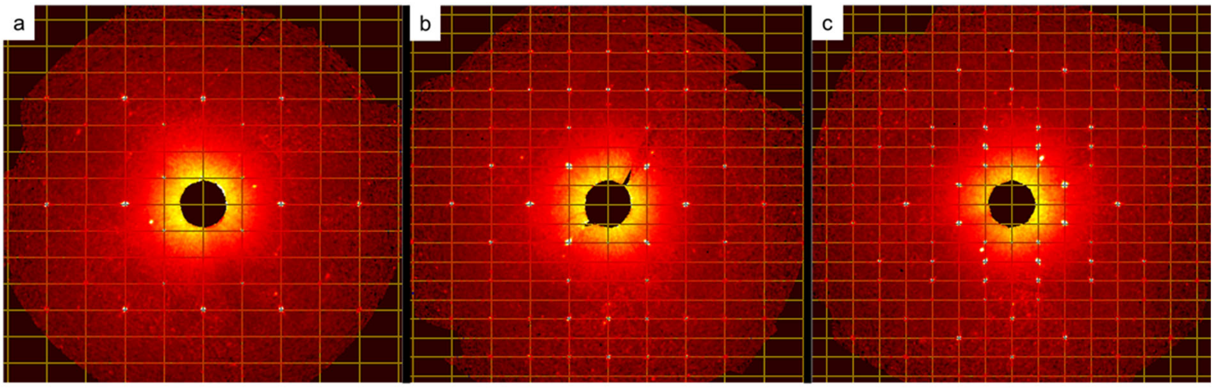

Figure S3. Diffraction pattern of CeGe with reciprocal lattice reconstructions of (**a**) (*hk*0), (**b**) (*h*0*l*), and (**c**) (0*kl*) layers.

3. Thermal Analysis

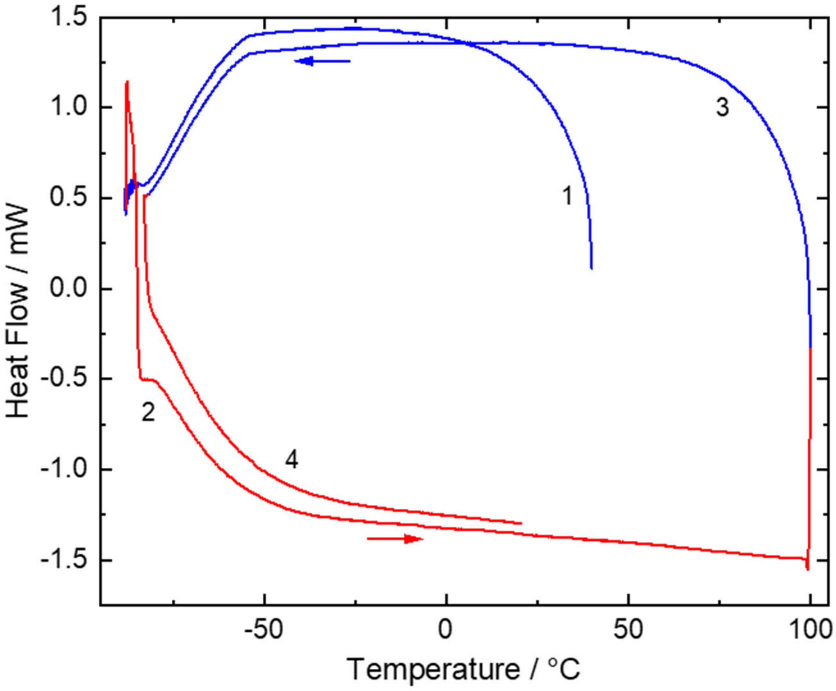

Figure S4. DSC, 1,3-first and second cooling, 2,4-first and second heating. Note the absence of any indication of a phase transformation in the interval measured.

4. Distances in Ge-polyanionic Motifs

Table S2. Binary alkaline earth and rare earth metal germanides comprising Ge-polyanions. Average distances are given for chain motifs.

Structure Type	Compound	Connectivity	Structural Motif	Distance in Chain	Ref.
Ca_7Sn_6	Ca_7Ge_6	(1*b*); (1*b*) + (2*b*)	Two layer types: dumbbells; chain fragments	2.549	[4]
	Sr_7Ge_6			2.569	[5]
	Ba_7Ge_6			2.571	[5]
CrB	CaGe	(2*b*)	infinite trans-trans chains	2.592	[6]
	SrGe			2.622	[7]
SrSi		(1*b*)+(2*b*) + (3*b*)	planar Si_{10} units	-	[8]
CrB	BaGe	(2*b*)	infinite trans-trans chains	2.638	[7]
FeB	LaGe			2.667	[9]
LaSi		(2*b*)	infinite cis-trans- cis-tans chains	2.621; 2.799	[10]
FeB	CeGe	(2*b*)	infinite trans-trans chains	2.674	this work
	PrGe			2.649	[11]
CrB				2.67	[11]
	NdGe			2.711	[11]
	SmGe			2.65	[12]
	EuGe			2.60	[13]
	GdGe			2.632	[12]
	TbGe			2.666	[14]
	DyGe			2.551	[15]
	HoGe			2.639	[16]
	ErGe			2.668	[11]
	TmGe			2.571	[17]
FeB	LuGe			2.597	[18]

Ba_3Ge_4	Ba_3Ge_4	(2*b*)+(3*b*)	isolated butterflies + trans-trans butterfly-chains	2.60	[19]
Er_3Ge_4	Gd_3Ge_4	(1*b*) + (2*b*)	chain fragments	2.632	[20]
α-$GdSi_{2-x}$	$LaGe_{1.6}$	(3*b*)	network	2.449	[21]
$TbGe_2$	$TbGe_2$	(2*b*)+(3*b*),(4*b*)	two layer types: zig-zag chains + Si_4-square nets	2.446	[22]
$ZrSi_2$(-defect)	$TmGe_2$	(2*b*),(4*b*)		2.544	[23]
	$LuGe_{2(-x)}$			2.683	[24]
Y_3Ge_5	Y_3Ge_5	(3*b*)	interconnected chains	2.560	[25]
Y_3Ge_5-defect	Sm_3Ge_5			2.556	[26]
	Gd_3Ge_5			2.5625	[26]
	Tb_3Ge_5			2.566	[27]
	Ho_3Ge_5			2.546	[28]
$DyGe_3$	YGe_3	(2*b*), (5*b*)	two types of layers: Chains + double sheets	2.72	[29]
$DyGe_3$-defect ("$Y(Ga_{0.13}Ge_{0.87})_{3.34}$")	$PrGe_{3+x}$			2.544	[30]
	$NdGe_{3+x}$			2.575	[30]
$DyGe_3$	$TbGe_3$			2.547	[31]
	$DyGe_3$			2.505	[32]
	$HoGe_3$			2.535	[33]
	$ErGe_3$			2.579	[34]
	$TmGe_3$			2.541	[32]
	$LuGe_3$			2.585	[35]

References

1. Hohnke, D.; Parthe, E. AB compounds with Sc, Y and rare earth metals. II. FeB and CrB type structures of monosilicides and germanides. *Acta Crystallogr*. **1966**, *20*(4), 572-582. https://doi.org/10.1107/S0365110X66001282
2. Haschke, H.; Nowotny, H.; Benesovsky, F. Untersuchungen im System Cer-Silicium-Germanium. *Monatsh. Chem*. **1966**, *97*(5), 1452-1458. https://doi.org/10.1007/BF00902596
3. Das, P.K.; Kumar, N.; Kulkarni, R.; Dhar, S.K.; Thamizhavel, A. Anisotropic magnetic properties and superzone gap formation in CeGe single crystal. *J. Phys.: Condens. Matter*, **2012**, *24*(14), 146003. https://doi.org/10.1088/0953-8984/24/14/146003
4. Palenzona, A.; Manfrinetti, P.; Fornasini, M.L. The phase diagram of the Ca-Ge system. *J. Alloys Compd*. **2002**, *345*, 144-147. https://doi.org/10.1016/S0925-8388(02)00326-2
5. Siggelkow, L.; Hlukhyy, V.; Fässler, T.F. Sr_7Ge_6, Ba_7Ge_6 and Ba_3Sn_2 –Three new binary compounds containing dumbbells and four-membered chains of tetrel atoms with considerable Ge–Ge π-bonding character. *J. Solid State Chem*. **2012**, *191*, 76-89. https://doi.org/10.1016/j.jssc.2012.03.008
6. Eckerlin, B.; Meyer, H.J.; Woelfel, E. Die Kristallstruktur von CaSn and CaGe. *Z. Anorg. Allg. Chem*. **1955**, *281*, 322-328. https://doi.org/10.1002/zaac.19552810508
7. Rieger, W.; Parthe, E. Alkaline earth silicides, germanides and stannides with CrB structure type. *Acta Crystallogr*. **1967**, *22*(6), 919-922. https://doi.org/10.1107/S0365110X67001793
8. Eisenmann, B.; Schaefer, H.; Turban, K. On a new SrSi-modification and the new compound SrGe0.76. *Z. Naturforsch. B* **1974**, *29*, 464-468. https://doi.org/10.1515/znb-1974-7-803
9. Duerr, I.; Bauer, B.; Roehr, C. Lanthan-Triel/Tetrel-ide La (Al, Ga)(x) (Si, Ge)(1-x). Experimentelle und theoretische Studien zur Stabilitaet polarer 1:1-Phasen. *Z. Naturforsch. B* **2011**, *66*(11), 1107-1121. https://doi.org/10.1515/znb-2011-1105
10. Mattausch, H.J.; Oeckler, O.; Simon, A. Eine neue Modifikation von Lanthanmonosilicid -IT-LaSi. *Z. Anorg. Allg. Chem*. **1999**, *625*, 1151-1154. https://doi.org/10.1002/(SICI)1521-3749(199907)625:7<1151::AID-ZAAC1151>3.0.CO;2-4
11. Schobinger Papamantellos, P.; Buschow, K.H.J. Ferromagnetism of NdGe and PrGe studied by neutron diffraction and magnetic measurements. *J. Less Common Met*. **1985**, *111*, 125-138. https://doi.org/10.1016/0022-5088(85)90178-X

12. Tharp, A.G.; Smith, G. S.; Johnson, Q. Structures of the rare earth germanides at or near equiatomic proportions. *Acta Crystallogr.* **1966**, *20*(4), 583-585. https://doi.org/10.1107/S0365110X66001294
13. Merlo F. Fornasini M.L. CrB-type equiatomic compounds of europium, ytterbium and alkaline-earth metals with Si, Ge, Sn, Pb. *J. Less-Common Met*. **1967**, *13*, 603-610. https://doi.org/10.1016/0022-5088(67)90105-1
14. Schobinger Papamantellos P. Buschow K.H.J. A neutron diffraction and magnetic study of the first-order phase transition in $TbGe_{1-x}Si_x$ (0 = x = 0.4). *J. Magn. Magn. Mater*. **1986**, *62*, 15-28. https://doi.org/10.1016/0304-8853(86)90729-8
15. Buschow K.H.J. Schobinger Papamantellos P. Fischer P. Magnetic structure and properties of equiatomic rare earth germanides. *J. Less-Common Met*. **1988**, *139*, 221-231. https://doi.org/10.1016/0022-5088(88)90003-3
16. Schobinger Papamantellos, P.; Buschow, K.H.J. Magnetic structure and incommensurate phase transition in HoGe. *J. Magn. Magn. Mater.* **1984**, *44*, 149-157. https://doi.org/10.1016/0304-8853(84)90058-1
17. Eremenko, V.N.; Meleshevich, K.A.; Buyanov, Yu.I.; Martsenyuk, P.S. Structure of the alloys and phase diagram of the thulium-germanium system. *Powder Metall. Met. Ceram*. **1989**, *28*, (7) 543-547. https://doi.org/10.1007/BF00794867
18. Freccero, R.; Hübner, J.-M.; Prots, Y.; Schnelle, W.; Schmidt, M.; Wagner, F. R.; Schwarz, U.; Grin, Y. "Excess" electrons in LuGe. *Angew. Chem. Int. Ed.* **2021**, *60*, 6457. https://doi.org/10.1002/anie.202014284
19. Zürcher, F.; Nesper, R. Ba_3Ge_4: Polymerization of Zintl Anions in the Solid and Bond Stretching Isomerism. *Angew. Chem*. **1998**, *37* (23), 3314-3318. https://doi.org/10.1002/(SICI)1521-3773(19981217)37:23<3314::AID-ANIE3314>3.0.CO;2-L
20. Tobash, P. H.; DiFilippo, G.; Bobev, S.; Hur, N.; Thompson, J. D.; Sarrao, J. L. Structure and properties of Gd_3Ge_4: the orthorhombic RE_3Ge_4 structures revisited (*RE* = Y, Tb-Tm). *Inorg. Chem*. **2007**, *46*, 8690– 8697. https://doi.org/10.1021/ic7009034
21. Guloy, A.M.; Corbett, J.D. Syntheses and structures of lanthanum germanide, $LaGe_{2-x}$, and lanthanum aluminum germanide, LaAlGe: interrelationships among the α-ThSi2, α-GdSi2, and LaPtSi structure types. *Inorg. Chem*. **1991**, *30*, 4789-4794. https://doi.org/10.1021/ic00025a021
22. Schobinger Papamantellos, P.; de Mooij, D.B.; Buschow, K.H.J. Crystallographic and magnetic structure of $TbGe_2$. *J. Less Common Met*. **1988**, *144*, 265-274. https://doi.org/10.1016/0022-5088(88)90140-3
23. Tobash, P.H.; Meyers, J.J.; DiFilippo, G.; Bobev, S.; Ronning, F.; Thompson, J.D.; Sarrao, J.L. Structure and properties of a new family of nearly equiatomic rare-earth metal-tin-germanides $RESn_{1+x}Ge_{1-x}$ (*RE*= Y, Gd-Tm): an unusual example of site preferences between elements from the same group. *Chem. Mater*. **2008**, *20* (6), 2151-2159. https://doi.org/10.1021/cm7033799
24. Francois, M.; Venturini, G.; Malaman, B.; Roques, B. Nouveaux isotypes de $CeNiSi_2$ dans les systemes *R-M-X* (*R* ident to La-Lu, *M* ident to metaux des groupes 7 A 11 et *X* ident to Ge, Sn). I compositions et parametres cristallins *J. Less Common Met*. **1990**, *160*, (2), 197-213. https://doi.org/10.1016/0022-5088(90)90381-S
25. Bruskov, V. A.; Bodak, O. I.; Pecharskii, V. K.; Gladyshevskii, E. I.; Muratova, L. A. Crystal structure of Y_3Ge_5 (''$YGe_{1.7}$''). *Kristallografiya* **1983**, *28* (2), 260– 263.
26. Tobash, P.H.; Lins, D.; Bobev, S.; Hur, N.; Thompson, J.D. Sarrao, J.L. Vacancy Ordering in $SmGe_{2-x}$ and $GdGe_{2-x}$ (x = 0.33): Structure and Properties of Two Sm_3Ge_5 Polymorphs and of Gd_3Ge_5. *Inorg. Chem*. **2006**, *45*, 7286-7294. https://doi.org/10.1021/ic060913f
27. Schobinger-Papamantellos, P.; de Mooij, D.B. Buschow, K.H.J. Crystallographic and magnetic structure of Dy_3Ge_5 and $DyGe_{1.9}$. *J. Less-Common Met*. **1990**, *163*, 319-330. https://doi.org/10.1016/0022-5088(90)90598-E
28. Zaharko, O.; Schobinger Papamantellos, P.; Ritter, C. Antiferromagnetic ordering in the novel Ho_3Ge_5 and $HoGe_{1.85}$ compounds studied by X-ray and neutron diffraction. *J. Alloys Compd*. **1998**, *280*, 4-15.
29. Belyavina, N. M.; Markiv, V. Ya.; Speka, M. V. Crystal structure of YGe_3, $YGe_{1.9}$ and a novel Y_3Ge_4 compound. *J. Alloys Compd.* **1999**, *283*, 162– 168, https://doi.org/10.1016/S0925-8388(98)00858-5
30. Fukuoka, H.; Yoshikawa, M.; Baba, K.; Yamanaka, S. Preparation and Structures of Lanthanoid Germanides, $PrGe_{3.36}$, $NdGe_{3.25}$, and $TmGe_3$ with Double Square Ge-Mesh Structures. *Bull. Chem. Soc. Jpn*. **2010**, *83*, 323–327. https://doi.org/10.1246/bcsj.20090310
31. Schobinger-Papamantellos, P., Andre, G., Rodriguez-Carvajal, J., de Groot, C.H., Boer, F.R de and Buschow, K.H.J. The magnetic ordering of the novel compound $TbGe_3$. *J. Alloys and Compd.* **1996**, 232, 165-168.

32. Schobinger-Papamantellos, P.; de Mooij, D. B.; Buschow, K. H. J. Crystal structure of the compound $DyGe_3$. *J. Alloys Compd.* **1992**, *183*, 181– 186. https://doi.org/10.1016/0925-8388(92)90743-S
33. Schobinger-Papamantellos, P.; Rodriguez Carvajal, J.; Tung, L.D.; Ritter, C.; Buschow, K.H.J. Competing multiple-q magnetic structures in $HoGe_3$: I. The magnetic phase diagram of $HoGe_3$. *J. Phys. Condens. Matter* **2008**, *20,* 195201. https://doi.org/10.1088/0953-8984/20/19/195201
34. Eremenko, V.N.; Obushenko, I.M. Phase Diagram of the Erbium-Germanium System. *Sov. Non-Ferrous Met. Res*. **1981**, *9*, 216–220.
35. Hübner, J.-M.; Bobnar, M.; Akselrud, L.; Prots, Y.; Grin, Y.; Schwarz, U. Lutetium Trigermanide $LuGe_3$: High-Pressure Synthesis, Superconductivity, and Chemical Bonding. *Inorg. Chem*. **2018**, *57*, 10295–10302. https://doi.org/10.1021/acs.inorgchem.8b01510
